# Force-exerting perpendicular lateral protrusions in fibroblastic cell contraction

**DOI:** 10.1038/s42003-020-01117-7

**Published:** 2020-07-21

**Authors:** Abinash Padhi, Karanpreet Singh, Janusz Franco-Barraza, Daniel J. Marston, Edna Cukierman, Klaus M. Hahn, Rakesh K. Kapania, Amrinder S. Nain

**Affiliations:** 1 Department of Mechanical Engineering, Virginia Tech, Blacksburg, VA USA; 2 Department of Aerospace and Ocean Engineering, Virginia Tech, Blacksburg, VA USA; 3 Cancer Biology Program, Marvin & Concetta Greenberg Pancreatic Cancer Institute, Fox Chase Cancer Center, Philadelphia, PA USA; 4 Department of Pharmacology, The University of North Carolina at Chapel Hill, Chapel Hill, NC 27599 USA

**Keywords:** Cellular motility, Biotechnology, Cancer microenvironment

## Abstract

Aligned extracellular matrix fibers enable fibroblasts to undergo myofibroblastic activation and achieve elongated shapes. Activated fibroblasts are able to contract, perpetuating the alignment of these fibers. This poorly understood feedback process is critical in chronic fibrosis conditions, including cancer. Here, using fiber networks that serve as force sensors, we identify “3D perpendicular lateral protrusions” (3D-PLPs) that evolve from lateral cell extensions named twines. Twines originate from stratification of cyclic-actin waves traversing the cell and swing freely in 3D to engage neighboring fibers. Once engaged, a lamellum forms and extends multiple secondary twines, which fill in to form a sheet-like PLP, in a force-entailing process that transitions focal adhesions to activated (i.e., pathological) 3D-adhesions. The specific morphology of PLPs enables cells to increase contractility and force on parallel fibers. Controlling geometry of extracellular networks confirms that anisotropic fibrous environments support 3D-PLP formation and function, suggesting an explanation for cancer-associated desmoplastic expansion.

## Abstract (web summary)

Padhi et al. employ nanofibers with controlled structure and alignment as an extra-cellular matrix model, on which they study the exertion of forces from adherent fibroblasts. Identifying force exerting 3D perpendicular lateral protrusions, authors describe a mechanism which leads to the contraction of parallel, neighbouring fibers, and the forces needed to move and align the neighbouring fibers. These findings have relevance in understanding cancer-associated desmoplastic expansion.

## Introduction

During acute wound healing, myofibroblastic activation of fibroblastic cells imparts inward forces and contracts the collagen-rich extracellular matrix (ECM). Upon wound resolution, scars are formed at the end of a process known as nemosis. Myofibroblastic cell contraction is pivotal to any physiological processes (e.g., developmental, acute wound healing), but in the absence of complete nemosis, it is also key to pathological instances like chronic inflammation and fibrosis-related diseases, including the microenvironmental desmoplasia of solid epithelial cancers^1,2^. It is thus not surprising that cancers have been described as chronic wounds for which desmoplasia, the contractile fibrous-like tumor-microenvironment, plays a pivotal role^3,4^. In solid epithelial tumors, desmoplasia expands in a manner that simulates chronic wound extension^5^. In fact, in extreme cases such as pancreatic ductal adenocarcinomas (PDAC), desmoplasia expands to encompass the majority of the tumor mass^6^. Interestingly, just as scarred tissue often presents with aligned collagen signatures, aligned ECM fibers were described as “tumor-associated collagen” or TAC signatures, hallmarks of detrimental patient outcomes^7–10^. Importantly, myofibroblastic cancer-associated fibroblasts (CAFs) are known to be the local activated cells responsible for the production and remodeling of the topographically aligned, anisotropic, desmoplastic ECM. Of note, we previously demonstrated that anisotropic ECMs generated by CAFs can activate naive fibroblastic cells into CAFs^11^, suggesting that aligned ECMs generated by CAFs could drive desmoplastic expansion. Despite extensive data regarding the role of aligned ECM in polarized migration^8,12–16^, very little is known about ECM-dependent desmoplastic expansion. It is commonly understood that in aligned ECMs, cell elongation and polarity are maintained due to minimal probing in lateral directions^15^. In such scenarios, it is not understood how cells stretch onto multiple-fibers, and how lateral protrusions contract inwards to aid in desmoplastic remodeling and expansion. This study identifies and characterizes a new type of contractile, lateral fibroblast protrusion through which aligned ECM triggers naive fibroblastic cell contraction, akin to myofibroblastic cells, thus promoting desmoplastic expansion.

Lateral protrusions or spikes are well-documented in multiple cell types (growth cones, mesenchymal, fibrosarcoma, hepatocytes, and conjunctival cells)^17,18^ and occur in varying sizes and morphologies to serve unique purposes^18,19^. These structures have been implicated in the proteolytic degradation of the 3D interstitial ECM by cancer cells^18^, and by leukocytes in probing the vascular membrane for permissive sites, which eventually allow vascular extravasation^20^. Leukocytes experiencing shear flow ‘roll,’ via transient tethering, using receptor-ligand bonds^21,22^. The tethering interactions are facilitated by microvilli shown to be 80 nm in diameter and varying in length from 350 nm to 1 μm or longer^23,24^. Similarly, lateral protrusions formed in melanoma cells have been documented to provide traction in a 3D environment in the absence of adhesion^25^. It was recently shown that spindle-shaped cells in 3D gel matrices maintain cell polarity and directed migration by forming limited numbers of lateral protrusions^26^, which are attributed to restriction in α_5_β_1_-integrin to the leading edge^15,27^, and activating RhoA laterally to inhibit Rac1-induced protrusions^27–29^. Overall, the utility of lateral protrusions remains poorly understood, as classic 2D culturing methods limit the formation of lateral protrusions, while 3D gels lack the homogeneity and topographic patterning needed to study the role of fiber geometry on protrusion frequency, morphology, and dynamics. To partially remedy this, we recently used orthogonal arrangement of fibers of mismatched fiber diameters to constrain cell migration along large diameter fibers while studying lateral protrusions of various shapes and sizes on smaller diameter fibers^30,31^. We observed the protrusions formed in an integrin-dependent manner on small diameter fibers, and notably suspended flat ribbons of equivalent widths did not capture protrusive sensitivity to curvature^30^. Here, using aligned and suspended fiber nanonets that also act as force sensors^32–34^, we report the identification of force exerting side protrusions termed ‘3D perpendicular lateral protrusions (3D-PLPs)’ that do not require pre-existing fibers to extend upon. We quantitatively show that anisotropic ECMs, represented by suspended and aligned nanonets as well as CAF derived ECMs, promote production of PLPs to enable fibroblastic cell contraction, potentially explaining the single cell/ECM physical characteristics of desmoplastic expansion.

## Results

### Twines from actin-ruffles engage neighboring fibers

**Fig. 1:**
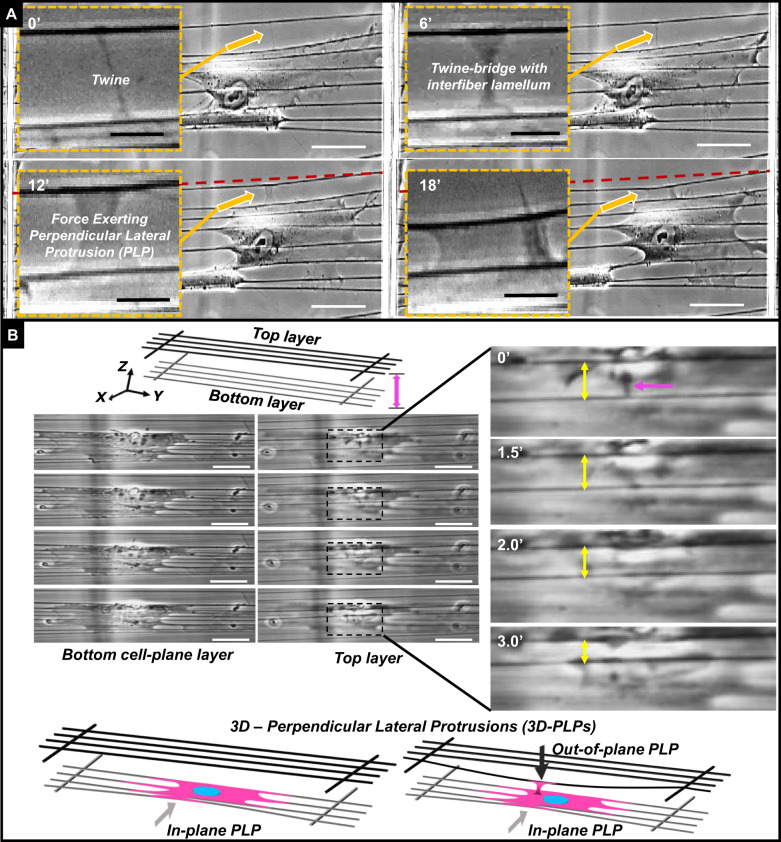
Transition from primary twines to 3D-perpendicular lateral protrusions (3D-PLPs). **a** Insets show magnified images of the sections indicated by arrows. The dashed red line shows the original, undeflected position of an extracellular fiber neighboring the cell. Time-lapse images of single cell in elongated shape spreading laterally through force exerting PLPs on neighboring fibers. A single twine contacts a neighboring fiber (upper left; 0′), leading to production of a second twine. The space between them is filled in to form a twine-bridge (upper right; 6′). Twines mature to become capable of exerting inward contractile forces at 12 min and proceed to mature and deflect further by 18 min (bottom images; 12′ and 18′, compare neighboring fiber deflection vs. red dotted line, marking the initial neighboring fiber position). **b** Twines (pink arrow) were also detected engaging fiber layers suspended above the cell plane (in this example the two-layer separation is 6 µm). After engagement, these twines developed into protrusions that were able to considerably deflect fibers of the top layer, pulling them inwards toward the cell body (decreasing length of yellow arrow). See accompanying movies M1 and M2. Scale bar: **a** 50 μm, inset 10 μm, **b** 20 μm.

On suspended and aligned fibers, using single layered or double stacked extracellular fiber arrays, we observed that elongated cells formed filamentous lateral protrusions (primary twines) that swung freely in 3D space until they effectively attached to neighboring ECM fibers (located on the side (Fig. 1a) or on top (Fig. 1b) of the main cell body). Subsequently, we observed initiation and formation of secondary twines that transitioned to formation of a twine-bridge, composed of primary and secondary twines, oriented perpendicular to cell body. We observed a sheet-like structure formed to fill the gap between the twines to form “3D-perpendicular lateral protrusions” (3D-PLPs, Supplementary Movies 1, 2). The PLPs exerted forces that enabled the inwards deflection of these neighboring fibers, in a manner that is suggestive of a cellular contraction oriented perpendicular to the main cell body axis.

**Fig. 2:**
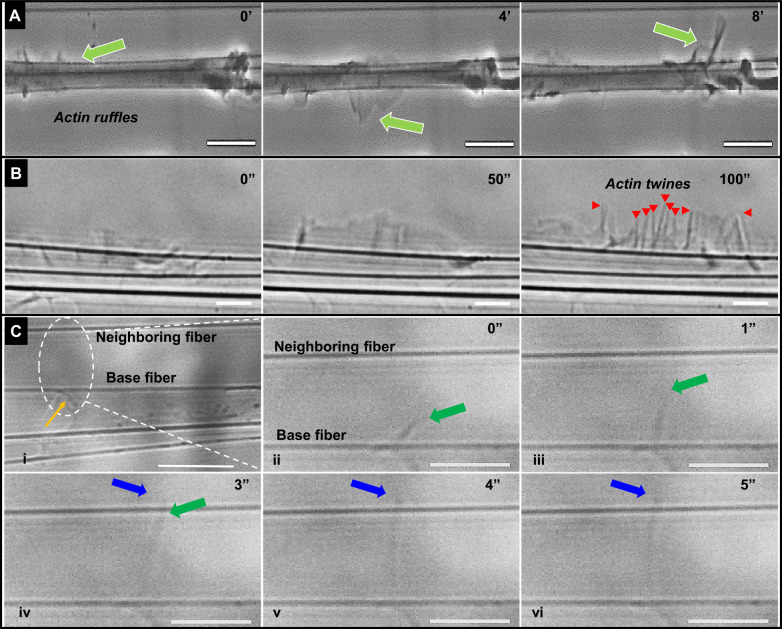
In anisotropic substrates, twines emerge from actin ruffles and engage with neighboring fibers. Time-lapse images showing (**a**) actin wave (green arrow) traveling along the length of the cell body (Movie M7). **b** Ruffles from the wave develop into striated structures that evolve into nano-filamentous nascent ‘twines’ shown by red arrowheads (Movie M8). **c** (i) Image of a cell with a primary twine pointed by the yellow arrow. (ii–vi) Time-lapse images of area indicated by dashed oval demonstrating that twine engagement with neighboring fiber takes place in ~2 s of intial primary twine contacting a neighboring fiber ((iv–vi), Movie M9). The green arrow shows growth of primary twine from cell body and blue arrow shows twine growth after engagement with neighboring fiber i.e. overhanging part of twine. Scale bar: **a** 20 μm, **b** 5 μm, **c** 5 μm.

To quantitatively describe the formation of 3D-PLPs, we inquired if lateral protrusions could form in the elongated cells, and asked if these exhibited a characteristic morphology that was associated with a specific cell geometry. For this, we used the previously described non-electrospinning spinneret based tunable engineered parameters (STEP^35,36^) method to fabricate anisotropic/aligned fibers. These could also serve as force sensing nanonets, as we described previously^32–34^. We followed, in real-time, the protrusions of different cell lines and proceeded to examine which behaviors, if any, were unique to fibroblastic cells (immortalized murine C2C12, immortalized embryonic fibroblast (MEF), and well-established NIH-3T3) and human mesenchymal stem cells (hMSCs) as well as Hela cells (Supplementary Fig. 1 and Supplementary Movies 3–6). All cell types acquired an elongated cell body, as expected, while continuously forming twines. The twines were located lateral to the long axis of the cell and found to originate from apparent membrane ruffles that resembled previously described cyclic actin waves^37^. The actin-rich membrane ruffles spiraled about the fiber axis (Fig. 2a, Supplementary Fig. 2, and Supplementary Movie 7) and advanced at 6.58 μm/min (*n* = 30), similar to the actin polymerization rates reported in the literature (7.20–8.73 μm/min)^38^. The ruffles extended beyond the main cell body and stratified into denser independent twines (Fig. 2b and Supplementary Movie 8) presumably due to folding-over and buckling^39^. The twines were found to grow in length and swing freely (in 3D) similar to reported angular rotations of filopodia^40–43^, which in turn enabled engagement with nearby neighboring extracellular fibers. The attachment to neighboring fibers occurred rapidly following contact between the twine and the neighboring fiber (Fig. 2c and Supplementary Movie 9).

### Steps in formation of 3D-perpendicular lateral protrusions

**Fig. 3:**
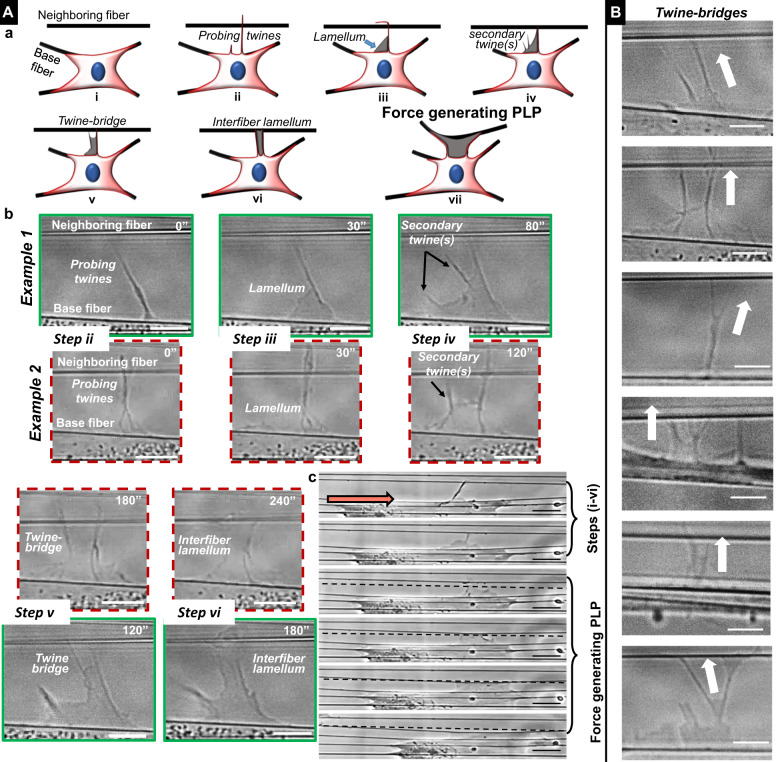
Discreet steps in the formation of force exerting perpendicular lateral protrusions (PLPs). **a** (a) Cartoons showing the process of forming force-exerting perpendicular lateral protrusions (PLPs). Cells attached to fibers form filamentous twines that engage with neighboring fiber (i). Subsequently, actin lamellum grows along the base of twine from cell body (ii) followed by formation of secondary twines (iii). Engagement of secondary twine with neighboring fiber leads to formation of a suspended twine-bridge (iv) that facilitates advancement of the lamellum that generates a suspended interfiber lamellum (v). Over time (~minutes), the interfiber lamellum broadens and applies contractile forces causing neighboring fiber to contract inwards, thus creating force exerting PLPs (vii). (b) Phase images in green and red (dashed) boxes depict two sample cases of PLPs in steps (i–vi). (c) Phase images of whole-cell forming PLP. Black dashed lines indicate undeflected position of neighboring fiber. Orange arrow indicates cell migration direction. **b** Sample images of twine-bridges of varying sizes and shapes and arrows point toward the neighboring fiber. Time in seconds. Scale bar: **a** (b) 5 μm, (c) 20 μm, **b** 5 μm.

We characterized the evolution of individual primary twines into 3D-PLPs. Using real-time phase microscopy of living cells, we observed the formation of primary twines, secondary twines, and twine-bridges (Supplementary Movie 10). The maturation of twines into force-exerting 3D-PLPs occurred in seven characteristic steps (Fig. 3a, (panel a, b)): Step (i): cells stretch, on anisotropic extracellular fibers (‘base fibers’), and achieve an elongated morphology parallel to the fiber orientation. Step (ii): the base fibers serve as anchors enabling the cell to probe for neighboring fibers by extending rigid (see below) “probing twines”; lateral protrusions that lunge outwards and sway from their base to encounter and engage with the neighboring fiber (Fig. 2c). Step (iii): cell extends a triangular lamellum anchored between the primary twine and the base fiber. Step: (iv) from the newly developed lamellum, one or more ‘secondary twines’ are formed similar to folding and buckling driven stratification of membrane ruffle described in Fig. 2. Step (v): secondary twines are defined and reach toward the neighboring fiber. When at least one secondary twine makes contact and adheres to the neighboring fiber, which has a primary twine attached to it, the two twines act as anchors, and the original lamellum begins to fill in the space between the main cell body at the base fiber and the neighboring fiber, thus forming a suspended ‘twine-bridge’ (Fig. 3b). Step (vi): after the twine-bridge completely fills the space between the two twines, the cell body (at the base fiber) and the neighboring fiber (forming an ‘interfiber lamellum’), the twine-bridge widens with a classic hour-glass shape to attain an early PLP (timepoint 6 minutes in Fig. 1). Step (vii): as the PLP widens/matures, it applies inward contractile forces, thus deflecting the neighboring fiber. The original neighboring fiber can now serve as a new base fiber, thus perpetuating inward fibroblastic cellular contraction. Surprisingly, we found that these seven steps in PLP formation were not isolated occurrences and importantly, were unique occurrences detected on cells cultured onto aligned suspended fiber networks (see below).

### Aligned extracellular fibers support force exerting 3D-PLPs

**Fig. 4:**
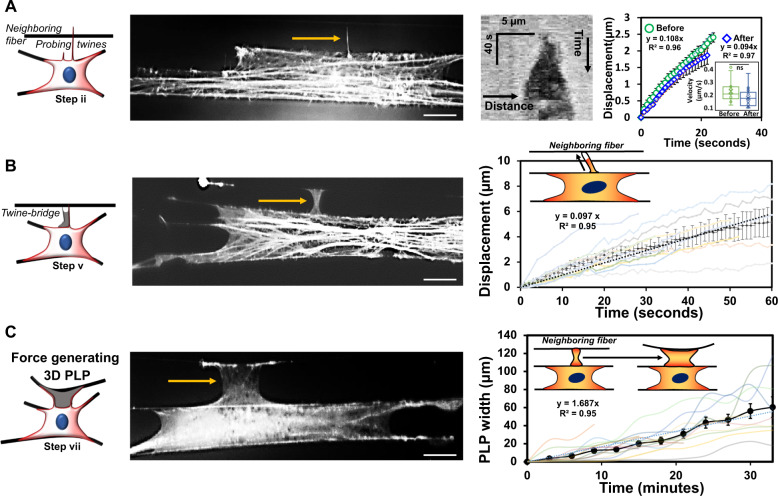
Actin kinetics in PLP formation. **a** Twines attached to neighboring fibers grow in width along the neighboring fiber axis as demonstrated by the kymograph. Kymograph was obtained by performing a line scan along the fiber axis. The average displacement vs. time profiles show twine advancement before and after engagement with neighboring fibers (*n* = 17 for both). The inset shows average velocity for the two categories. **b** Displacement vs. time profiles showing extension rates of actin lamellum between twine-bridges (*n* = 13). Inset shows cartoon of actin lamellum advancement along the twine-bridge. **c** Broadening of twine-bridge into force exerting PLP (*n* = 15). Linear fits to average of all profiles is included in each plot. In **a**–**c** representative immunostained f-actin images are included to show distinct geometries of twines and 3D-PLPs. Scale bar 20 microns.

Filopodial extensions and their transition to lamellipodial structures are well-known to be driven by actin dynamics at the leading edge. We inquired if the twine-PLP transition was also actin-based (Fig. 4). We recorded the growth rate of primary twines as they emerged from actin-rich ruffles and found them to extend at ~0.1 μm/s, which matches the reported kinetics of filopodia extension rates (~0.12–0.2 μm/s^44^). After engagement with neighboring fibers, the primary twines continued to grow at rates similar to those prior to engagement (inset in Fig. 4a), yet often maintained the attachment point with the neighboring fiber. Next, we analyzed the growth of lamellae to form the twine-bridges and found that they advance at the rate of ~0.1 μm/s (Fig. 4b), similar to actin polymerization rates (~0.12–0.14 μm/s)^38^. We saw that twine-bridges could mature into PLP structures that exert contractile force as they broadened. We measured the width of twine-bridges halfway along their span length and found them to widen at ~0.028 μm/s (Fig. 4c), which matched the actin retrograde flow rates in the lamellipodial tip (~0.008–0.025 μm/s) but were much faster than flow rates reported in lamella (~0.004–0.008 μm/s)^45^. Overall, our measurements of twine, lamellum, and twine-bridge widening rates indicate that these potentially actin-based structures are detected in highly specific sequence of morphological changes and are often associated with specific geometry. Thus, we speculate that the observed particular twine-PLP transitions are similar to other known, actin-based filopodial-lamellipodial transitions.

**Fig. 5:**
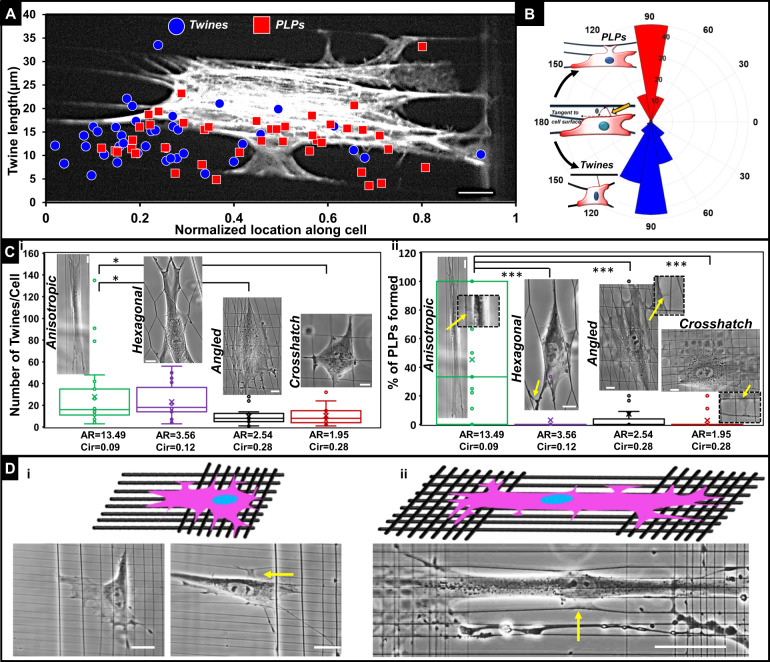
Twine and PLPs. **a** Plot showing the length of twines observed with respect to corresponding location of emergence of twines and PLPs along the normalized cell length. Backdrop shows a cell forming multiple lateral protrusions. **b** Polar histogram of angle formed by twine or PLP engagement axis and a tangent to cell body (*n* = 84 for PLP and 123 for twines). PLPs are oriented mostly perpendicularly to cell body. **c** Four fiber network designs were created to interrogate cell shape (AR: aspect ratio and Circ.: circularity) driven number density of (i) twines, and (ii) PLP formation ((number of PLPs/number of attached twines) *100). Elongated cells of high aspect ratios and small circularities form maximum number of twines/cell on anisotropic and hexagonal networks, while only anisotropic networks facilitate formation of PLPs. Data are analyzed from 23 randomly selected videos for each category. **d** Design of fiber networks (i) crosshatch interfaced with aligned, and (ii) crosshatch networks on either side of aligned networks to demonstrate that 3D-PLPs are formed only in aligned networks (yellow arrows). On crosshatch networks cells form classicly described protrusions mostly along existing fibers. Scale bar: **a** 10 µm, **c** 10 µm, **d** (i) 20 µm, (ii) 50 µm.

We noted that twines and PLPs of varying lengths (5–35 µm) were evident along the entire length of the cell body, as opposed to being localized mostly at the leading edge of cells (Fig. 5a). Intriguingly, we found that twines were attached to neighboring fibers over a wide range of angles, but the ensuing PLPs were primarily oriented orthogonal to the parent cell body (Fig. 5b). We also inquired if the spatial organization of twines and PLPs were specific to cells attached to anisotropic fiber networks. We constructed fiber networks with three new designs (hexagonal, angled, and with crosshatches) and recorded the number of twines (length ≥3 µm) forming in each fiber category. We found that the greatest twine formation per cell over a two-hour period (imaged every two minutes) occurred in elongated cells with high aspect ratios (observed more often on anisotropic and hexagonal structures, Fig. 5c(i)). Since twines were attaching to neighboring fibers in all experimental conditions, we calculated the number of PLPs formed from twines attached to neighboring fibers (Fig. 5c(ii)). Not surprisingly, we found that, on average, nearly half of engaged twines transitioned to PLPs in cells that had spread onto anisotropic parallel fibers, whereas for all other fiber network/substrates, we rarely encountered the formation of PLPs. To confirm that aligned extracellular fibers used to simulate the anisotropic fiber architecture of desmoplastic ECMs, were primed environments for inducing cells to form 3D-PLPs, we designed two new mixed network/substrates (Fig. 5d): crosshatch networks transitioning to aligned fibers (Supplementary Movie 11), and aligned fibers having crosshatch networks on either end (Supplementary Movie 12). Cells on crosshatch networks formed classic protrusions mainly along the existing fibers, while 3D-PLPs (shown by yellow arrows) were evident solely at areas of the substrate that included the aligned fiber networks. Altogether, these data demonstrate that the maximum number of actin-based twines are formed in elongated cell shapes, but the transition of these twines to force-exerting PLPs almost exclusively occurs in anisotropic fiber arrangements.

### Forces exerted by twine-bridges and 3D-PLPs

It is now well-appreciated that cells in vivo and in 3D interstitial ECM exert contractile forces on the neighboring fibrous environments either by pushing or tugging individual fibers^18,46,47^. We observed that twines that transitioned to broad PLP structures, resembling lamellipodia, were able to pull neighboring fibers inwards toward the cell body, allowing cells to spread on multiple fibers, while maintaining a main cell body axis matching that of the aligned fiber orientation. Thus, we inquired how forces developed in 3D-PLPs, and also investigated the resultant migratory and force response of cells as they spread from three to four and five fibers using 3D-PLPs.

**Fig. 6:**
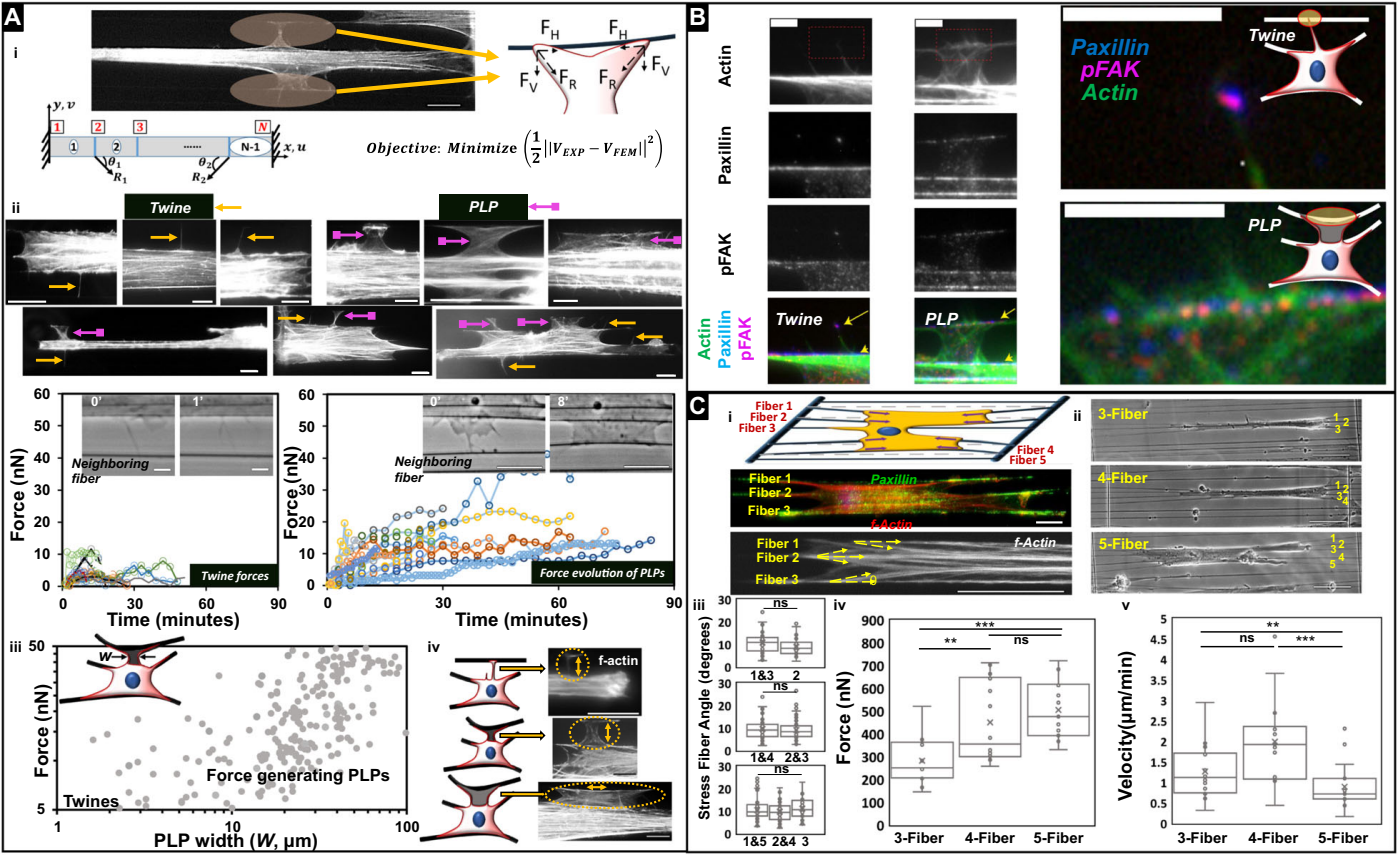
Forces applied by *PLPs* facilitate cell spreading. **a** (i) Representative images and schematic of forces exerted by twine-bridges. Force vectors are assumed to act along the membrane curvature with origin at paxillin cluster lengths. The force vectors R_1_ and R_2_ are calculated by minimizing the objective function, where V_EXP_ represents the experimental displacement profile of the fixed-fixed beam and V_FEM_ is the prediction from the finite element model. (ii) Representative immunostained images for actin localization in twines and PLPs of varying sizes (top panel) and both structures formed simultaneously in the same cell (bottom panel). Force response of twines and PLPs with corresponding images. (iii) Forces in developing PLPs increase beyond 10 µm width (W) shown in the cartoon (*n* = 25 PLPs). (iv) f-actin stress fibers transition from being along the direction of developing PLPs to along the neighboring fibers in developed PLPs. **b** Left-panel: f-actin stress fibers, paxillin and pFAK in twines and PLPs show transition from classic focal adhesions in twines to elongated 3D-adhesions in PLPs. Right-panel shows magnified color images regions shown by yellow arrows in left-panel and signifying highlighted regions in the cartoon. **c** (i) Schematic representation of NFM method to measure contractile forces of single cells attached to multiple fibers. Force vectors originate from paxillin focal adhesion clustering (green) and act along f-actin stress fibers (red and white). (ii) Representative phase images of anisotropically stretched cells on 3, 4, and 5 fibers. iii F-actin stress fiber angle data used in measurement of forces: fiber 1 and 3 (*n* = 53, *n* signifying number of stress fibers) and fiber 2 (*n* = 30) in 3-fiber category, fiber 1 and 4 (*n* = 80) and fiber 2 and 3 (*n* = 84) in 4-fiber category, fiber 1 and 5 (*n* = 72), fiber 2 and 4 (*n* = 70), and fiber 3 (*n* = 35) in 5-fiber category. iv NFM based average force calculated for 3 time points separated by 3 min each for each cell (*n* = 13, 14, and 15 for 3, 4, and 5 fiber categories, respectively). v. Migration rates for each fiber category (*n* = 15 in each). Scale bar: **a** (i) 10 µm, (ii) 10 μm, (iv) 10 μm, **b** 5 μm, **c** (i) 20 μm, (ii) 50 μm.

First, we quantitated the forces exerted by both twines and PLPs using nanonet force microscopy (NFM^32–34^, Fig. 6a(i)) through the establishment of tension-bearing force vectors directed toward the cell body. Specifically, the vectors originate at the paxillin positive attachment sites and point along the membrane curvature in twine-bridges and PLPs. For twine-bridges less than 6 µm in width, a single force vector pointing vertically toward the parent cell body was assigned, as the twines and twine-bridges enriched in actin was pointed along their direction (Fig. 6a(ii)). For PLPs wider than 6 μm, two force vectors on either side of twine-bridges were assigned, as the dominant actin stress fibers were formed along the membrane curvature. We classified the ability of twines to exert force if they deflected the neighboring fibers ≥2 μm, which for the fibers used in this study corresponds to ~2 nN in resolution. Forces were estimated using an optimization framework that minimized the difference between experimental data (measured fiber displacement profile) and custom finite element model prediction (Fig. 6a(i))^33^. Our analysis revealed that twines angled from the parent cell body could apply forces for only a brief period, which prevented their maturation into broad lamellipodial PLP structures. However, we found that twines could transition to force exerting PLPs when the angle transitioned close to 90 degrees due to the movement/shifting of the cell body. Force generation increased with twine-bridge width (W, measured at the middle of span length and shown in the cartoon in Fig. 6a(iii)), and for PLPs of widths greater than 10 µm, we observed a sharp increase in force generation, presumably due to well-defined organization of actin stress fibers and their alignment with the parent cell body. Overall, our force analysis indicates that transition of f-actin stress fiber orientation from orthogonal to in line with cell elongation axis indicates that a neighboring fiber (Fig. 6a(iv)) can become a new base fiber for further cell spreading, while inward cell contraction, pulling the extracellular fibers toward the cell body, is achieved. Immunostaining for f-actin, paxillin, and pFAK revealed that twines comprised of f-actin stress fibers while paxillin and pFAK localized at neighboring fiber attachment sites (shown by arrows, Fig. 6b). The listed molecular composition and orientation of these sites denote classic focal adhesion-like structures^48^. Once 3D-PLPs are established onto the neighboring fibers, the newly formed elongated cell-matrix structures resemble 3D-adhesions; include f-actin stress fibers that run along the direction of cell body (parallel to ECM anisotropy) and are enriched with paxillin^48,49^. Further, pFAK enrichment at PLPs constitutes an indication of a myofibroblastic/pathologic type of 3D-adhesions that was reported in desmoplasia^50^.

Next, since cells were using *PLPs* in anisotropic cell migration to spread onto neighboring fibers, we inquired how the inside-out contractile state of cells changed when they exert force and actively spread from 3 to 4 and 5 fibers (Fig. 6c(i, ii)). For the calculation of forces applied by cells, we approximated a strategy of using a single force vector that originated at the site of paxillin focal adhesion clustering (FAC) and pointed along the dominant f-actin stress fiber (Fig. 6c(i)). We chose this strategy because our previous studies indicated that cells attached to ~250 nm diameter fibers formed focal adhesion clusters (FAC) at the poles^32^. Overall, stress fibers were found on average to be angled between 8–11 degrees (Fig. 6c(iii)). We quantified the transient change in stress fiber angles as cells migrated and observed that stress fibers maintain their relative positions (Supplementary Fig. 3). The overall contractile force was computed by summation of the magnitudes of individual forces acting at the adhesion clusters ($F_{{\rm{Cell}}} = {\sum} {F_{{\rm{FAC}}}}$). Cells spread on 5 fibers exerted higher forces (~505 nN), and as a consequence had statistically lower migration rates compared to those on 3 (~282 nN) or 4 fibers (~451 nN) (Fig. 6c(iv, iv)). We found that cells attached to fibers had a symmetric distribution of forces with the highest contractility driven by fibers at the outermost boundaries of cells (Supplementary Fig. 4).

## Discussions

It has long been thought that scarring ECMs, evident in wound healing, and desmoplastic fibrotic ECMs in cancer share similar features, and well-established findings point to fibroblastic-to-myofibroblastic activation as key culprits. In contrast, the physiologically normal stroma includes ECMs that are naturally randomly oriented and tumor suppressive^4,51,52^. We have previously shown that cell-derived ECMs (also known as CDMs) are physiologically relevant 3D culturing systems^49,53^. Importantly, we demonstrated that CAF CDMs are anisotropic^11^ and, just like shown by the late P Keely, CAF-CDMs and CAF expressed biomarkers are predictive of bad outcomes in patients^8,50,54^. Further, we reported that CAF CDMs impart a naive-to-CAF myofibroblastic activation^11^. Hence, the detection of 3D-PLP structures in naive fibroblasts cultured within anisotropic CAF CDMs (Supplementary Fig. 5 and Supplementary Movies 13 and 14), constitutes strong evidence of the pathophysiological relevance of 3D-PLPs as well as that of the bioengineered anisotropic system presented in this study.

Strategies to ablate the culprit CAFs have indeed reduced stroma, yet offered no benefit to pancreatic ductal adenocarcinoma (PDAC) patients^55^, or in some cases even dangerously promoted cancer progression^56,57^. Thus, there is growing appreciation for “stroma normalization”, i.e. reinstitution of physiological ECM, and not its elimination, that is expected to impart clinical benefit^51,58–62^. A direct route to achieve stroma normalization is first understanding the biophysical links that perpetuate desmoplastic expansion, and second developing informed intervention strategies that dissuade this expansion. In this study, we describe a new functional role of anisotropic environments, which enable naive fibroblasts to become increasingly elongated and contract, akin to activated myofibroblastic cells, thus continually fueling desmoplastic expansion. We show for the first time to the best of our knowledge that fibroblastic cells can form contractile force-generating three dimensional perpendicular lateral protrusions (3D-PLPs) anywhere along the length of their body that contract inwards; evident by measurable neighboring fiber deflection.

3D-PLPs are formed in an orchestrated chain of events starting from actin ruffles that give rise to primary twines, which resemble microspikes and filopodia. Twines lunge and attach to neighboring fibers in a matter of seconds, which is in agreement with a recent study showing fibroblasts adhering to fibronectin in ≤5 s in an α5β1 integrin-dependent manner^63^. While microspikes and filopodia rarely exceed lengths of ~10 μm^64^, the lengths of the twines observed here had a wide distribution (a short ~5 μm to an extremely long ~35 μm). To the best of our knowledge, filopodia of such long lengths have been reported only in sea urchin embryos^65^. Filopodia in cells cultured on 2D substrates localize to the leading edge, and can transition to broad lamellipodial structures. Filopodia-lamellipodial transitions that occur at sides of cells cultured in 2D are usually associated with cell turning during migration. In contrast, we discovered filopodia-like twines, formed in cells cultured onto (and within) anisotropic extracellular fibers, are oriented orthogonal to the parent cell, and their transition to lamellipodial PLP does not trigger a change in direction of cell migration. Instead, 3D-PLPs apply inward contractile forces resulting in an overall elongated cell shape over an increased number of “pulled inward” fibers (see Supplementary Movie 15 for cell spreading from four to five aligned fibers without losing the main axis of the cell).

The parallel fibers used in our method mimic the aligned ECMs produced by CAFs in vitro, and the TACs_2/3_ fiber configurations in vivo, known to enable CAF activation^11,50^. The inter-fiber spacing (~10 µm) used in our study mimics those measured by the fibroblastic CDM models and that of second harmonic generation (SHG) of polarized light desmoplastic imaging of patient samples^7,9,50,53,66,67^. In vitro, at larger inter-fiber spacing^68,69^, cells attach to single fibers in spindle morphologies with limited instances of twine formation, akin to the behavior reported in bio-engineered aligned 3D collagen^15^. However, the formation of 3D-PLP was similar to those observed in cells that spread on multiple fiber/substrates. Since twines were capable of freely swinging in 3D, we fabricated fiber networks composed of two layers of aligned fibers, one on top of the other. We found that the twine from the bottom layer attached to a fiber in the top layer and was able to pull the neighboring extracellular fiber downwards and inwards toward the parent cell body (Fig. 1d and Supplementary Movie 2), thus suggesting a similarity in desmoplastic expansion in all directions in aligned CAF CDMs (Supplementary Movies 13, 14). We also used three different fiber homogenous architectures (Fig. 5c): hexagonal, angled and crosshatched, and heterogenous architectures (crosshatches interfaced with aligned networks, Fig. 5d) to determine the role of network designs in the formation of 3D-PLPs. Parallel and hexagonal networks supported persistent cell migration in elongated shapes, while angled and crosshatched networks induce spread shapes and random migration. Elongated cell shapes on hexagonal and parallel networks form the maximum number of twines that can engage with neighboring fibers. However, only parallel (i.e., anisotropic) fibers have the propensity to support formation of force-generating PLPs. In heterogeneous architectures, cells formed protrusions along existing crosshatch networks while 3D-PLPs formed only as cells either migrated onto aligned fibers or in regions of cell body already stretched along the intermediary aligned fibers. Thus, we emphasize that ECM architecture and elongated cell shape are key physical determinants of PLP formation that is potentially needed for naive-to-myofibroblastic activation to enable desmoplastic expansion.

The parallel fibers also served as force sensors. While individual filopodia have been shown to exert forces(pN–nN)^70–74^, here using NFM we quantitated the transient force response of twines developing into broad 3D-PLPs capable of exerting higher forces (tens of nano newtons), thus providing the necessary support for cells to spread onto neighboring fibers. The increase in force generation in 3D-PLPs coincides with their increase in width (Fig. 6a(iii)), which is correlated with the presence and spatial organization of f-actin stress fibers. Correlating the alignment of f-actin stress fibers with activation of phosphorylated focal adhesion kinase (pFAK) at adhesion sites on neighboring fibers (magnified images in Fig. 6b) provides further evidence of in vivo-like CAF 3D-adhesion formation and cells being activated to become force-generating myofibroblasts^50^. We emphasize that our method of force measurement establishes force vectors originating from focal adhesion clustering (FAC) sites, akin to mature focal adhesions localized at the edge of f-actin filaments on both ends of the elongated cell, and directed along f-actin stress fibers, in contrast to other methods where the force vectors are determined independent of stress fiber orientation^75–77^.

In conclusion, using NFM, we show in a reproducible and replicable manner that elongated cells on anisotropic fiber networks promote features akin to desmoplasia while contracting through the formation of force-generating lateral protrusions. Our findings identify a new cell behavior that describes the missing biophysical link in the desmoplastic expansion of aligned/anisotropic (i.e., desmoplastic) ECM. Further study of the density (Supplementary Movie 16), organization, and size of fibers, coupled with RhoGTPase signaling in PLPs, may provide intervention strategies to deter matrix-driven fibrous spread in cancers and chronic wounding diseases.

## Methods

### Nanonet manufacturing and cell culture

The suspended polystyrene fibers were manufactured by the previously reported non-electrospinning STEP technique^35^. A network of small diameter parallel fibers (~250 nm) was deposited 8–10 µm apart upon a layer of base fibers (>1 μm) spaced 350 µm apart and fused at the intersections. The scaffolds were placed in glass-bottom 6-well plates (MatTek, Ashland, MA) and sterilized in 70% ethanol for 10 min. After two rounds of PBS wash, 50 μl of 4 μg/ml of Fibronectin (Invitrogen, Carlsbad, CA) was put on the scaffolds and incubated at 37 °C for 30 min to facilitate cell adhesion. Bone marrow-derived human mesenchymal stem cells, (hMSCs; Lonza Inc, Basel, Switzerland) were cultured in supplemented growth medium (Lonza Inc) at 37 °C and 5% CO_2_. Cells were seeded on the scaffolds by placing 50 μl droplets with cell density of 100,000 cells/ml for 1 hour and then 2ml of growth medium was added to each well. CAF-CDMs were produced as previously described by us^78^.

### Time-lapse microscopy and cell force calculations

Nanonets in 6 well plates were placed in an incubating microscope (AxioObserver Z1, Carl Zeiss, Jena, Germany). Time Lapse movies were created by taking images with 20× objective at 3 min or 40/63X at the 1-second interval with an AxioCam MRm camera (Carl Zeiss). All measurements were performed using AxioVision (Carl Zeiss) and ImageJ (National Institute of Health, Bethesda, MD). Using beam mechanics, cell forces were estimated from experimentally obtained deflection of fibers. Briefly, an optimization algorithm is written in MATLAB (MathWorks, Natick, MA) that matched the experimental and computational finite-element fiber deflections to calculate forces at each time point (for details on model development, optimization algorithm and validation tests see Supplementary information^33^).

### Immunohistochemistry and immunofluorescence imaging

Cells on fibers were fixed in 4% paraformaldehyde, permeabilized in 0.1% Triton X100 solution and blocked in 5% goat serum. Paxillin (Tyr31) staining was done using primary rabbit anti-paxillin antibodies (Invitrogen) at a dilution of 1:250 and incubated at 4 °C for 1 h. Secondary goat anti-rabbit Alexa Fluor 488 (Invitrogen) antibodies were incubated for 45 min at room temperature in the dark. For double immunostaining, total Paxillin (Host: Mouse, Invitrogen) primary antibodies at a dilution of 1:40 and Phospho-FAK (Tyr397) (Host: Rabbit, Invitrogen) primary antibodies at a dilution of 1:200 were used and incubated for 4 h. Secondary antibodies goat anti-mouse Alexa Fluor 647 and goat anti-rabbit Alexa Fluor 488 were used at dilution of 1:150 and incubated for 1.5 h at room temperature. F-Actin stress fibers were stained using Rhodamine Phalloidin (SantaCruz Biotechnologies). Cell nuclei were stained with 300 nM of DAPI (Invitrogen) for 5 min. The scaffolds were kept in 2 ml antifade imaging solution during imaging. Fluorescent images were taken using an Axio Observer Z1 microscope (Carl Zeiss). Actin live cell imaging was performed as per the manufacturer’s instructions on using the reagent CellLight Actin-RFP, Bacman 2.0 (Invitrogen).

### Statistics and reproducibility

Sample populations were tested for statistical significance using the Student’s *t*-test and analysis of variance (ANOVA) for multiple group comparisons in GraphPad software (GraphPad Prism, California). Error bars represent standard errors. Values are reported as an average ± 1 SE. * Denotes *p*-value ≤ 0.05, **p-value ≤ 0.01, and *** denotes *p*-value ≤ 0.001. Measurements were obtained from different samples with the sample sizes being mentioned in each figure caption. Experimental reproducibility was established by performing several independent experiments (>5 independent experiments performed on different days with different cell passages).

## Supplementary information

Supplementary Information

Supplementary Information 2

Supplementary Information 3

Supplementary Information 4

Supplementary Information 5

Supplementary Information 6

Supplementary Information 7

Supplementary Information 8

Supplementary Information 9

Supplementary Information 10

Supplementary Information 11

Supplementary Information 12

Supplementary Information 13

Supplementary Information 14

Supplementary Information 15

Supplementary Information 16

Supplementary Information 17

Supplementary Information 18

Supplementary Information 19

## Data Availability

All data are available in the excel sheets uploaded on Figshare with the link: https://figshare.com/s/cb88ba6c96c5c0c6aa93. Any remaining data that support the findings of this study are available from the corresponding author upon reasonable request.

## References

[1] Hinz B, Gabbiani G (2003). Mechanisms of force generation and transmission by myofibroblasts. Curr. Opin. Biotechnol..

[2] Hinz B, Mastrangelo D, Iselin CE, Chaponnier C, Gabbiani G (2001). Mechanical tension controls granulation tissue contractile activity and myofibroblast differentiation. Am. J. Pathol..

[3] Dvorak HF (1986). Tumors: wounds that do not heal. Similarities between tumor stroma generation and wound healing. N. Engl. J. Med..

[4] Rybinski B, Franco-Barraza J, Cukierman E (2014). The wound healing, chronic fibrosis, and cancer progression triad. Physiol. Genomics.

[5] Alexander J, Cukierman E (2016). Stromal dynamic reciprocity in cancer: intricacies of fibroblastic-ECM interactions. Curr. Opin. Cell Biol..

[6] Erkan M (2008). The activated stroma index is a novel and independent prognostic marker in pancreatic ductal adenocarcinoma. Clin. Gastroenterol. Hepatol..

[7] Conklin MW (2011). Aligned collagen is a prognostic signature for survival in human breast carcinoma. Am. J. Pathol..

[8] Goetz JG (2011). Biomechanical remodeling of the microenvironment by stromal caveolin-1 favors tumor invasion and metastasis. Cell.

[9] Bredfeldt JS (2014). Automated quantification of aligned collagen for human breast carcinoma prognosis. J. Pathol. Inform..

[10] Conklin MW (2018). Collagen Alignment as a Predictor of Recurrence after Ductal Carcinoma In Situ. Cancer Epidemiol. Biomark. Prev..

[11] Amatangelo MD, Bassi DE, Klein-Szanto AJP, Cukierman E (2005). Stroma-derived three-dimensional matrices are necessary and sufficient to promote desmoplastic differentiation of normal fibroblasts. Am. J. Pathol..

[12] Provenzano PP (2006). Collagen reorganization at the tumor-stromal interface facilitates local invasion. BMC Med..

[13] Provenzano PP, Inman DR, Eliceiri KW, Trier SM, Keely PJ (2008). Contact guidance mediated three-dimensional cell migration is regulated by Rho/ROCK-dependent matrix reorganization. Biophys. J..

[14] Heck JN (2012). Microtubules regulate GEF-H1 in response to extracellular matrix stiffness. Mol. Biol. Cell.

[15] Riching KM (2014). 3D collagen alignment limits protrusions to enhance breast cancer cell persistence. Biophys. J..

[16] Oudin MJ (2016). Tumor cell-driven extracellular matrix remodeling drives haptotaxis during metastatic progression. Cancer Discov..

[17] Adams JC (2001). Cell-matrix contact structures. Cell. Mol. Life Sci..

[18] Wolf K, Friedl P (2009). Mapping proteolytic cancer cell-extracellular matrix interfaces. Clin. Exp. Metastasis.

[19] Taylor AC, Robbins E (1963). Observations on microextensions from the surface of isolated vertebrate cells. Dev. Biol..

[20] Nourshargh S, Hordijk PL, Sixt M (2010). Breaching multiple barriers: Leukocyte motility through venular walls and the interstitium. Nat. Rev. Mol. Cell Biol..

[21] McEver RP, Zhu C (2010). Rolling Cell Adhesion. Annu. Rev. Cell Dev. Biol..

[22] Ramachandran V (2001). Dimerization of a selectin and its ligand stabilizes cell rolling and enhances tether strength in shear flow. Proc. Natl Acad. Sci. USA.

[23] Bruehl RE, Springer TA, Bainton DF (1996). Quantitation of L-selectin distribution on human leukocyte microvilli by immunogold labeling and electron microscopy. J. Histochem. Cytochem..

[24] Karnik R (2008). Nanomechanical control of cell rolling in two dimensions through surface patterning of receptors. Nano Lett..

[25] Tozluoǧlu M (2013). Matrix geometry determines optimal cancer cell migration strategy and modulates response to interventions. Nat. Cell Biol..

[26] Nelson CM, Bissell MJ (2006). Of Extracellular Matrix, Scaffolds, and Signaling: Tissue Architecture Regulates Development, Homeostasis, and Cancer. Annu. Rev. Cell Dev. Biol..

[27] Petrie RJ, Doyle AD, Yamada KM (2009). Random versus directionally persistent cell migration. Nat. Rev. Mol. Cell Biol..

[28] Worthylake RA, Burridge K (2003). RhoA and ROCK promote migration by limiting membrane protrusions. J. Biol. Chem..

[29] Pankov R (2005). A Rac switch regulates random versus directionally persistent cell migration. J. Cell Biol..

[30] Koons B (2017). Cancer protrusions on a tightrope: nanofiber curvature contrast quantitates single protrusion dynamics. ACS Nano.

[31] Mukherjee A, Behkam B, Nain AS (2019). Cancer cells sense fibers by coiling on them in a curvature-dependent manner. iScience.

[32] Sheets K, Wang J, Zhao W, Kapania R, Nain AS (2016). Nanonet force microscopy for measuring cell forces. Biophys. J..

[33] Tu-Sekine, B. et al. Inositol polyphosphate multikinase is a metformin target that regulates cell migration. *FASEB J*. **33**,14137–14146 (2019). 10.1096/fj.201900717RR PMC6894044 31657647

[34] Padhi A (2020). Bioenergetics underlying single-cell migration on aligned nanofiber scaffolds. Am. J. Physiol. Cell Physiol..

[35] Wang J, Nain AS (2014). Suspended micro/nanofiber hierarchical biological scaffolds fabricated using non-electrospinning STEP technique. Langmuir.

[36] Jana, A. et al. Crosshatch nanofiber networks of tunable interfiber spacing induce plasticity in cell migration and cytoskeletal response. *FASEB J*. **33**, 10618–10632 (2019). 10.1096/fj.201900131R PMC6766658 31225977

[37] Guetta-Terrier C (2015). Protrusive waves guide 3D cell migration along nanofibers. J. Cell Biol..

[38] Giannone G (2007). Lamellipodial actin mechanically links myosin activity with adhesion-site formation. Cell.

[39] Pontes B (2017). Membrane tension controls adhesion positioning at the leading edge of cells. J. Cell Biol..

[40] Zidovska A, Sackmann E (2011). On the mechanical stabilization of filopodia. Biophys. J..

[41] Tamada A, Kawase S, Murakami F, Kamiguchi H (2010). Autonomous right-screw rotation of growth cone filopodia drives neurite turning. J. Cell Biol..

[42] Albrecht-Buehler G (1976). Filopodia of spreading 3T3 cells: do they have a substrate-exploring function?. J. Cell Biol..

[43] Bornschlögl T (2013). How filopodia pull: what we know about the mechanics and dynamics of filopodia. Cytoskeleton.

[44] Mogilner A, Rubinstein B (2005). The physics of filopodial protrusion. Biophys. J..

[45] Tomasello M (2004). Two distinct actin networks drive the protrusion of migrating cells. Science.

[46] Hodor PG, Illies MR, Broadley S, Ettensohn CA (2000). Cell-substrate interactions during sea urchin gastrulation: Migrating primary mesenchyme cells interact with and align extracellular matrix fibers that contain ECM3, a molecule with NG2-like and multiple calcium-binding domains. Dev. Biol..

[47] Wyckoff JB, Pinner SE, Gschmeissner S, Condeelis JS, Sahai E (2006). ROCK- and myosin-dependent matrix deformation enables protease-independent tumor-cell invasion in vivo. Curr. Biol..

[48] Cukierman E, Pankov R, Yamada KM (2002). Cell interactions with three-dimensional matrices. Curr. Opin. Cell Biol..

[49] Cukierman E (2001). Taking cell-matrix adhesions to the third dimension. Science.

[50] Franco-Barraza J (2017). Matrix-regulated integrin αvβ5 maintains α5β1-dependent desmoplastic traits prognostic of neoplastic recurrence. Elife.

[51] Schnittert J, Bansal R, Prakash J (2019). Targeting pancreatic stellate cells in cancer. Trends Cancer.

[52] Park CC, Bissell MJ, Barcellos-Hoff MH (2000). The influence of the microenvironment on the malignant phenotype. Mol. Med. Today.

[53] Malik R (2019). Rigidity controls human desmoplastic matrix anisotropy to enable pancreatic cancer cell spread via extracellular signal-regulated kinase 2. Matrix Biol..

[54] Gupta V (2011). Elevated expression of stromal palladin predicts poor clinical outcome in renal cell carcinoma. PLoS ONE.

[55] Kim EJ (2014). Pilot clinical trial of hedgehog pathway inhibitor GDC-0449 (vismodegib) in combination with gemcitabine in patients with metastatic pancreatic adenocarcinoma. Clin. Cancer Res..

[56] Cannon A (2018). Desmoplasia in pancreatic ductal adenocarcinoma: insight into pathological function and therapeutic potential. Genes Cancer.

[57] Hirata E, Sahai E (2017). Tumor microenvironment and differential responses to therapy. Cold Spring Harb. Perspect. Med..

[58] Whittle MC, Hingorani SR (2019). Fibroblasts in pancreatic ductal adenocarcinoma: biological mechanisms and therapeutic targets. Gastroenterology.

[59] Sherman MH (2014). Vitamin D receptor-mediated stromal reprogramming suppresses pancreatitis and enhances pancreatic cancer therapy. Cell.

[60] Neesse A, Krug S, Gress TM, Tuveson DA, Michl P (2013). Emerging concepts in pancreatic cancer medicine: targeting the tumor stroma. Onco. Targets Ther..

[61] Neesse A (2019). Stromal biology and therapy in pancreatic cancer: ready for clinical translation?. Gut.

[62] Bigelsen S (2018). Evidence-based complementary treatment of pancreatic cancer: a review of adjunct therapies including paricalcitol, hydroxychloroquine, intravenous vitamin C, statins, metformin, curcumin, and aspirin. Cancer Manag. Res..

[63] Strohmeyer N, Bharadwaj M, Costell M, Fässler R, Müller DJ (2017). Fibronectin-bound α5β1 integrins sense load and signal to reinforce adhesion in less than a second. Nat. Mater..

[64] Saarikangas J (2009). Molecular Mechanisms of Membrane Deformation by I-BAR Domain Proteins. Curr. Biol..

[65] Mattila PK, Lappalainen P (2008). Filopodia: Molecular architecture and cellular functions. Nat. Rev. Mol. Cell Biol..

[66] Robinson BK, Cortes E, Rice AJ, Sarper M, Del Río Hernández A (2016). Quantitative analysis of 3D extracellular matrix remodelling by pancreatic stellate cells. Biol. Open.

[67] Novotny GEK, Gnoth C (1991). Variability of fibroblast morphology in vivo: a silver impregnation study on human digital dermis and subcutis. J. Anat..

[68] Sheets K, Wunsch S, Ng C, Nain AS (2013). Shape-dependent cell migration and focal adhesion organization on suspended and aligned nanofiber scaffolds. Acta Biomater..

[69] Sharma P (2017). Aligned fibers direct collective cell migration to engineer closing and nonclosing wound gaps. Mol. Biol. Cell.

[70] Bornschlogl T (2013). Filopodial retraction force is generated by cortical actin dynamics and controlled by reversible tethering at the tip. Proc. Natl Acad. Sci. USA.

[71] Cojoc, D. et al. Properties of the force exerted by filopodia and lamellipodia and the involvement of cytoskeletal components. *PLoS ONE* **2**, e1072 (2007). 10.1371/journal.pone.0001072 PMC2034605 17957254

[72] Mogilner A, Rubinstein B (2005). The physics of filopodial protrusion. Biophys. J..

[73] Hochmuth RM, Shao JY, Dai J, Sheetz MP (1996). Deformation and flow of membrane into tethers extracted from neuronal growth cones. Biophys. J..

[74] Bridgman PC, Dailey ME (1989). The organization of myosin and actin in rapid frozen nerve growth cones. J. Cell Biol..

[75] Beningo KA, Wang YL (2002). Flexible substrata for the detection of cellular traction forces. Trends Cell Biol..

[76] Steinwachs J (2016). Three-dimensional force microscopy of cells in biopolymer networks. Nat. Methods.

[77] Cesa CM (2007). Micropatterned silicone elastomer substrates for high resolution analysis of cellular force patterns. Rev. Sci. Instrum..

[78] Franco-Barraza, J., Raghavan, K. S., Luong, T. & Cukierman, E. in *Methods in Cell Biology* Vol.156, 109–160 (Academic Press Inc., 2020). 10.1016/bs.mcb.2019.11.014 PMC7298733 32222216

