# Author Correction: Force-exerting perpendicular lateral protrusions in fibroblastic cell contraction

**DOI:** 10.1038/s42003-020-01196-6

**Published:** 2020-08-21

**Authors:** Abinash Padhi, Karanpreet Singh, Janusz Franco-Barraza, Daniel J. Marston, Edna Cukierman, Klaus M. Hahn, Rakesh K. Kapania, Amrinder S. Nain

**Affiliations:** 1 Department of Mechanical Engineering, Virginia Tech, Blacksburg, VA USA; 2 Department of Aerospace and Ocean Engineering, Virginia Tech, Blacksburg, VA USA; 3 Cancer Biology Program, Marvin & Concetta Greenberg Pancreatic Cancer Institute, Fox Chase Cancer Center, Philadelphia, PA USA; 4 Department of Pharmacology, The University of North Carolina at Chapel Hill, Chapel Hill, NC 27599 USA

Correction to: *Communications Biology* 10.1038/s42003-020-01117-7, published online 21 July 2020.

In the original version of the published Article, there were errors in the placement of the black dashed lines in Fig. 3c. The errors affected the interpretation of the undeflected position of neighboring fibers. The figure has been replaced in the PDF and HTML versions of the Article.

