# Supplementary material for: Force-exerting perpendicular lateral protrusions in fibroblastic cell contraction
Source: Commun Biol. 2020 Jul 21;3:390. doi: 10.1038/s42003-020-01117-7 (PMC7374753; doi:10.1038/s42003-020-01117-7)
Supplement: Supplementary file 1 — Supplementary Information [file 42003_2020_1117_MOESM1_ESM.pdf]

Supplementary Figures

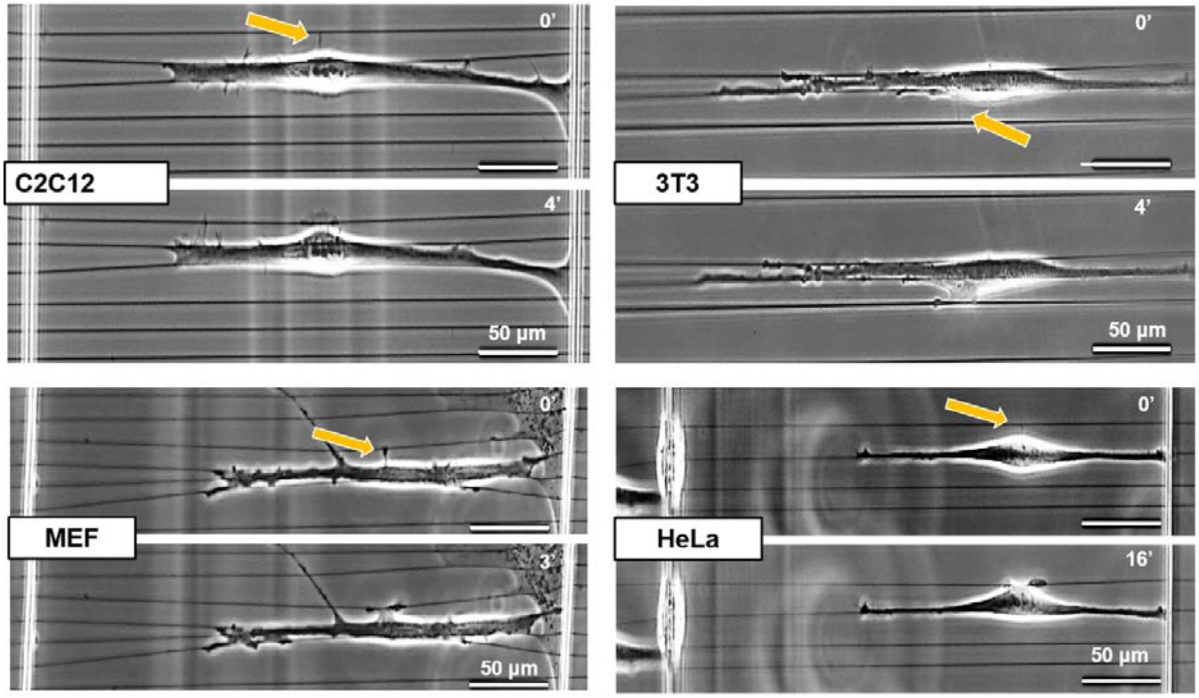

Supplementary Figure 1. Force exerting PLPs formed by different cell lines. PLP formation through twine engagement on neighboring fibers in anisotropic environments in four different cell lines including three naïve fibroblastic cells, C2C12, NIH-3T3 and MEFs, while hela cells show a mesenchymal type of cell spread.

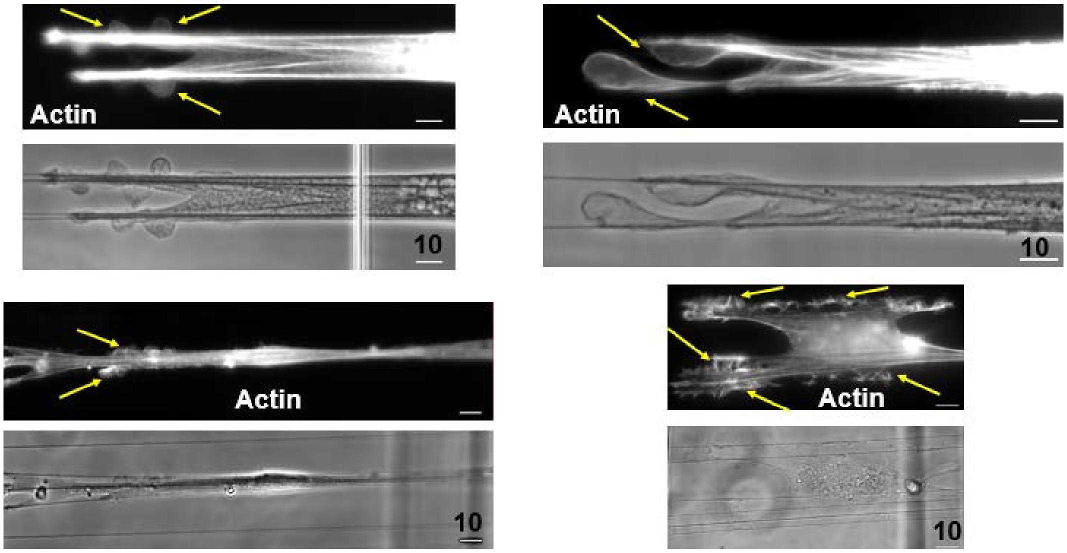

Supplementary Figure 2. Ruffles that form twines are rich in actin. Representative phase contrast and immunostained images for actin showing ruffles (yellow arrows) to be rich in actin. Ruffles form at multiple locations along the cell body. Scale bars are in microns.

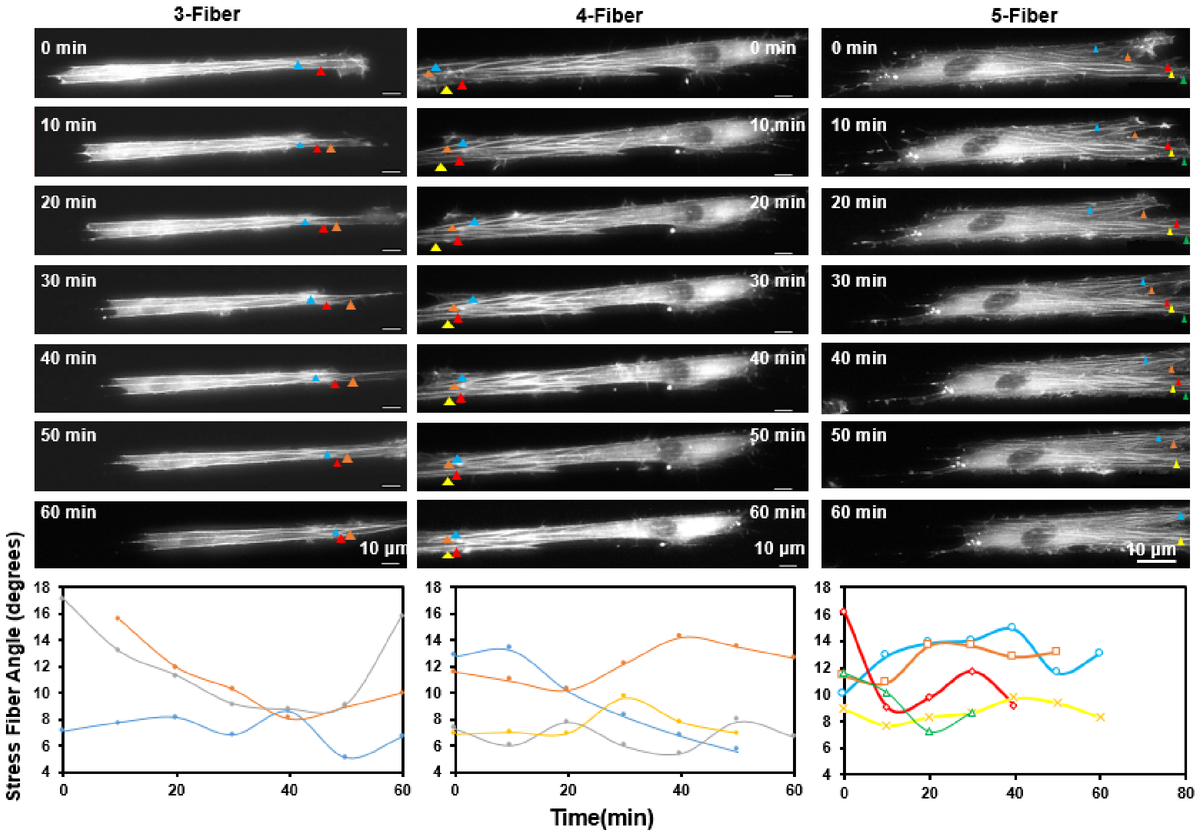

Supplementary Figure 3. Transient analysis of f-actin stress fibers in cells attached to 3, 4, and 5 fibers. Representative profiles of cells migrating on 3 and 4-fiber networks. Analysis shows that the stress fibers maintain their relative orientation with respect to fiber axes during cell migration.

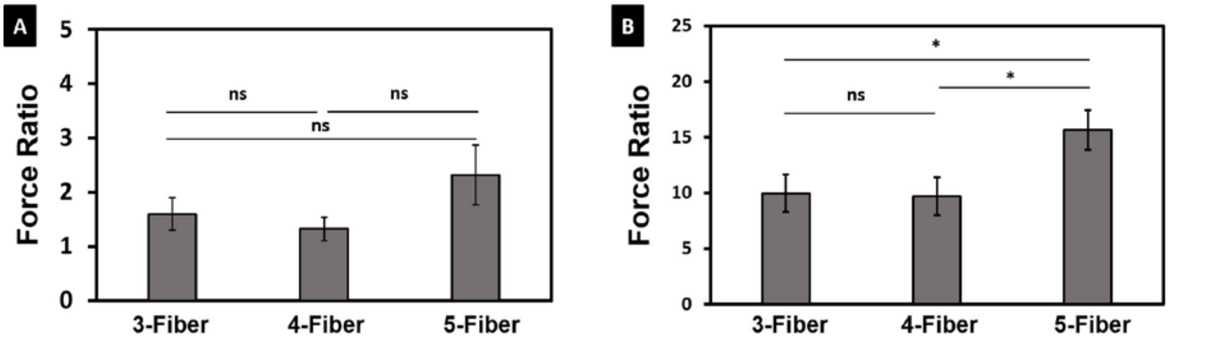

Supplementary Figure 4. Force distribution across a cell body for cell attached to 3, 4 and 5 fibers (A) Force ratios demonstrating symmetric distribution of forces: 3-Fiber- ($\frac{F1/F2}{F3/F2}$), 4-Fiber-($\frac{F1/F2}{F4/F3}$), 5-Fiber- ($\frac{F1/F2}{F5/F4}$) (B) Force ratios of forces at periphery to center of cell: 3-Fiber- (F1/F2), 4-Fiber- (F1/F2), 5-Fiber (F1/F3). F1 indicates force on Fiber-1, F2 force on Fiber-2, F3 force on Fiber-3, F4 force on Fiber-4, F5 force on Fiber-5. Numbering of fibers done from top to bottom (see figure 3 C(i) in main text). Sample size: n=13, 14 and 15 for 3, 4 and 5-fiber categories.

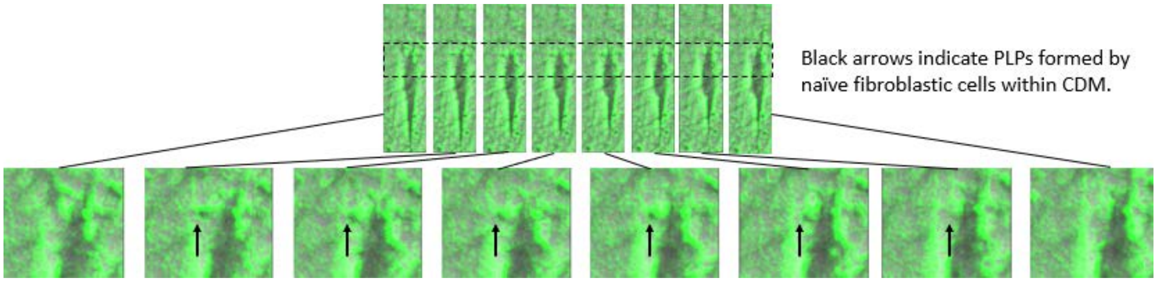

Supplementary Figure 5. Existence of 3D-PLPs (i.e., lateral protrusions) in naïve fibroblastic cells migrating within *in vivo* like desmoplastic environments. Time lapse images taken every fifteen minutes of a single naïve fibroblastic cell migrating in human pancreatic CAF-cell-derived matrices (CDMs). Lateral protrusions similar to 3D-PLPs are shown by black arrow.
