# Supplementary material for: Force-exerting perpendicular lateral protrusions in fibroblastic cell contraction
Source: Commun Biol. 2020 Jul 21;3:390. doi: 10.1038/s42003-020-01117-7 (PMC7374753; doi:10.1038/s42003-020-01117-7)
Supplement: Supplementary file 2 — Supplementary Information 2 [file 42003_2020_1117_MOESM2_ESM.pdf]

Descriptions of Additional Supplementary Files

Supplementary Movie 1: 3D-PLP formation in primary human mesenchymal stem cells (hMSC): Force exerting perpendicular lateral protrusions form through the engagement of twines on neighboring fiber that over time mature into twine bridges. Time is shown on top left in hours:minutes:seconds

Supplementary Movie 2: 3D-PLPs within two fiber layers: Synchronized timelapse videos of a cell attached to bottom fiber layer that forms a twine (black arrow, left movie), which engages with a fiber in the top layer (black arrow in right movie). Subsequently, the twine develops into 3D-PLP causing inward contraction of the fiber (white arrows). Time is shown on top left in hours:minutes: seconds

Supplementary Movie 3: 3D-PLPs in mouse myoblast C2C12: Lateral protrusion (shown by arrows) that develops into PLPs. Time is shown on top left in hours:minutes: seconds

Supplementary Movie 4: 3D-PLPs in mouse fibroblast 3T3: Lateral protrusion (shown by arrows) that develops into PLPs. Time is shown on top left in minutes:seconds

Supplementary Movie 5: 3D-PLPs in mouse embryonic fibroblast (MEF): Lateral protrusion (shown by arrows) that develops into PLPs. Time is shown on top left in hours:minutes:seconds

Supplementary Movie 6: 3D-PLPs in Human cervical tumor HeLa: Lateral protrusion (shown by arrows) that develops into PLPs. Time is shown on top left in hours:minutes:seconds

Supplementary Movie 7: Actin waves along cell body: Membrane ruffles spiral as actin wave about fiber axis. Time is shown on top left in hours:minutes:seconds

Supplementary Movie 8: Lateral twines form through membrane ruffles: Membrane ruffles extending from cell body stratify into denser structures termed twines. Time is shown on top left in seconds:thousandths

Supplementary Movie 9: Twine engagement with neighboring fibers: Twine engagement to neighboring fiber occurs in 1-2 s. Time is shown on top left in seconds:thousandths

Supplementary Movie 10: Establishment of twine-bridges: Growth of lamellum at the base of primary twine followed by the formation of secondary twine that facilitates establishment of primary-secondary twine-bridge. Time is shown on top left in seconds:thousandths

Supplementary Movie 11: 3D-PLP formation hetereogenous crosshatch-aligned fiber networks: 3D-PLP (black arrow) is formed when a cell transitions from crosshatch networks to aligned fibers. On crosshatch networks, protrusions are formed along existing fibers. Time is shown on top left in hours:minutes:seconds

Supplementary Movie 12: 3D-PLP formation in hetereogenous crosshatch-aligned-crosshatch fiber networks: A single cell attached to three distinct fiber configurations forms 3D-PLPs (black arrows) only

in the aligned fiber configuration. On crosshatch networks, protrusions (white arrows) are formed along existing fibers. Time is shown on top left in hours:minutes:seconds

Supplementary Movie 13: Live 3D-PLP base coming out of CAF generated CDM plane in a naïve fibroblasts. Zoomed 6 hour movie showing a frame per 15 minutes in which a naïve fibroblastic cell was cultured within a pancreatic CAF CDM. Note the lateral protrusions coming out of the cell body (shown by black arrows).

Supplementary Movie 14: Live 3D-PLP base coming out of CAF generated CDM plane in a naïve fibroblasts. Zoomed 6 hour movie showing a frame per 15 minutes in which a naïve fibroblastic cell was cultured within a pancreatic CAF CDM. Note the 3D swirling twine protrusion coming out of the cell body (white arrow).

Supplementary Movie 15: 3D-PLPs force exertion facilitates cell spreading: Cell attached to four parallel fibers forms 3D-PLPs (black arrows) that apply contractile forces, allowing cell to spread to five fibers. Time is shown on top left in hours:minutes:seconds

Supplementary Movie 16: Dynamic twines and 3D-PLPs formed on aligned multiple fibers: Migrating human mesenchymal stem cell attached to nine fibers forms symmetric shape and displays active twine dynamics and 3D-PLPs. Time is shown on top left in hours:minutes:seconds
