# Supplementary material for: Force-exerting perpendicular lateral protrusions in fibroblastic cell contraction
Source: Commun Biol. 2020 Jul 21;3:390. doi: 10.1038/s42003-020-01117-7 (PMC7374753; doi:10.1038/s42003-020-01117-7)
Supplement: Supplementary file 19 — Supplementary Information 19 [file 42003_2020_1117_MOESM19_ESM.pdf]

nature research

Corresponding author(s): Amrinder Nain (COMMSBIO-20-0001-T)

Last updated by author(s): Apr 28, 2020

Reporting Summary

Nature Research wishes to improve the reproducibility of the work that we publish. This form provides structure for consistency and transparency in reporting. For further information on Nature Research policies, see Authors & Referees and the Editorial Policy Checklist.

Statistics

For all statistical analyses, confirm that the following items are present in the figure legend, table legend, main text, or Methods section.

n/a	Confirmed	
☐	☒	The exact sample size (*n*) for each experimental group/condition, given as a discrete number and unit of measurement
☐	☒	A statement on whether measurements were taken from distinct samples or whether the same sample was measured repeatedly
☐	☒	The statistical test(s) used AND whether they are one- or two-sided<br>*Only common tests should be described solely by name; describe more complex techniques in the Methods section.*
☒	☐	A description of all covariates tested
☐	☒	A description of any assumptions or corrections, such as tests of normality and adjustment for multiple comparisons
☐	☒	A full description of the statistical parameters including central tendency (e.g. means) or other basic estimates (e.g. regression coefficient) AND variation (e.g. standard deviation) or associated estimates of uncertainty (e.g. confidence intervals)
☐	☒	For null hypothesis testing, the test statistic (e.g. *F*, *t*, *r*) with confidence intervals, effect sizes, degrees of freedom and *P* value noted<br>*Give P values as exact values whenever suitable.*
☒	☐	For Bayesian analysis, information on the choice of priors and Markov chain Monte Carlo settings
☒	☐	For hierarchical and complex designs, identification of the appropriate level for tests and full reporting of outcomes
☐	☒	Estimates of effect sizes (e.g. Cohen's *d*, Pearson's *r*), indicating how they were calculated

Our web collection on statistics for biologists contains articles on many of the points above.

Software and code

Policy information about availability of computer code

Data collection

Zeiss AxioObserver Z1 microscope was operated using AxioVision 4.8.2 software.

Data analysis

1. Captured time lapse movies were analyzed using AxioVision 4.8.2 and NIH ImageJ (1.8.0_66) for length and angle measurements.
2. Forces were calculates using custom code written in MATLAB 2017a code. Further details about the analytical model can be found in the supplementary section of our previously published paper (Tu-Sekine, B., Padhi, A., Jin, S., Kalyan, S., Singh, K., Apperson, M., Kapania, R., Hur, S.C., Nain, A. and Kim, S.F., 2019. Inositol polyphosphate multikinase is a metformin target that regulates cell migration. The FASEB Journal, 33(12), pp.14137-14146.)
3. Graphad Prism 8 was used for statistical analysis.
4. Microsoft Excel 2016 was used for calculations and plotting.

For manuscripts utilizing custom algorithms or software that are central to the research but not yet described in published literature, software must be made available to editors/reviewers. We strongly encourage code deposition in a community repository (e.g. GitHub). See the Nature Research guidelines for submitting code & software for further information.

Data

Policy information about availability of data

All manuscripts must include a data availability statement. This statement should provide the following information, where applicable:

- Accession codes, unique identifiers, or web links for publicly available datasets
- A list of figures that have associated raw data
- A description of any restrictions on data availability

The data that support the findings of this study are available from the corresponding author upon reasonable request.

Field-specific reporting

Please select the one below that is the best fit for your research. If you are not sure, read the appropriate sections before making your selection.

☒ Life sciences ☐ Behavioural & social sciences ☐ Ecological, evolutionary & environmental sciences

For a reference copy of the document with all sections, see nature.com/documents/nr-reporting-summary-flat.pdf

Life sciences study design

All studies must disclose on these points even when the disclosure is negative.

Sample size	Sample size was determined based on previous studies from our group.
Data exclusions	We have not explicitly excluded any data from our analysis. However, we set predetermined criteria for analysis which have been reported either in the methods section or the results section of the manuscript.
Replication	Data collection was done over multiples rounds of experiments.
Randomization	Selection of videos to be analyzed were chosen randomly from sets of experiments.
Blinding	Blinding was not essential in our study as measurements reported in manuscript are of physical nature.

Reporting for specific materials, systems and methods

We require information from authors about some types of materials, experimental systems and methods used in many studies. Here, indicate whether each material, system or method listed is relevant to your study. If you are not sure if a list item applies to your research, read the appropriate section before selecting a response.

Materials & experimental systems

n/a	Involved in the study	
☐	☒	Antibodies
☐	☒	Eukaryotic cell lines
☒	☐	Palaeontology
☒	☐	Animals and other organisms
☒	☐	Human research participants
☒	☐	Clinical data

Methods

n/a	Involved in the study	
☒	☐	ChIP-seq
☒	☐	Flow cytometry
☒	☐	MRI-based neuroimaging

Antibodies

Antibodies used	Paxillin, Invitrogen (Cat#AHO0492, Lot#UF2787562A)<br>Phospho-FAK, Invitrogen (Cat#44624G, Lot#2160101)<br>Rhodamine Phalloidin, Santa Cruz Biotechnology (Cat#sc-301530, Lot#C2619)<br>Alexa Fluor 488, Invitrogen (Cat#A11034, Lot#1124089)<br>Alexa Fluor 647, Invitrogen (Cat#A21235, Lot#1939631)<br>CellLight Actin-RFP, BacMam 2.0, Invitrogen (Cat#C10502, Lot#1985346)
Validation	Paxillin and Phospho-FAK have been validated for application in immunohistochemistry and immunofluorescence as stated on company website and have associated citations where the products have been previously used. Certificate of analysis is available from companies upon request.

Eukaryotic cell lines

Policy information about cell lines

Cell line source(s)	Adult bone marrow derived human mesenchymal stem cells, Lonza Inc, Basel, Switzerland
Authentication	Cell line was authenticated by Lonza Inc. as specified in the certificate of analysis provided on their website.
Mycoplasma contamination	Cells were mycoplasma free as tested by Lonza and were used as received.
Commonly misidentified lines (See ICLAC register)	No commonly misidentified cell lines were used in this study.
